# Three-Dimensional Bioprinting of Cartilage by the Use of Stem Cells: A Strategy to Improve Regeneration

**DOI:** 10.3390/ma11091749

**Published:** 2018-09-17

**Authors:** Livia Roseti, Carola Cavallo, Giovanna Desando, Valentina Parisi, Mauro Petretta, Isabella Bartolotti, Brunella Grigolo

**Affiliations:** RAMSES Laboratory, Rizzoli RIT-Research, Innovation & Technology Department, Istituto di Ricerca Codivilla Putti, IRCCS Istituto Ortopedico Rizzoli, Via di Barbiano, 1/10, 40136 Bologna, Italy; carola.cavallo@ior.it (C.C.); giovanna.desando@ior.it (G.D.); parisi_valentina@hotmail.it (V.P.); mauro.petretta@ior.it (M.P.); isabella.bartolotti@ior.it (I.B.); brunella.grigolo@ior.it (B.G.)

**Keywords:** bioink, 3D bioprinting, biomaterials, stem cells, cartilage, meniscus, osteoarthritis, 4D printing, organ-on-a-chip

## Abstract

Cartilage lesions fail to heal spontaneously, leading to the development of chronic conditions which worsen the life quality of patients. Three-dimensional scaffold-based bioprinting holds the potential of tissue regeneration through the creation of organized, living constructs via a “layer-by-layer” deposition of small units of biomaterials and cells. This technique displays important advantages to mimic natural cartilage over traditional methods by allowing a fine control of cell distribution, and the modulation of mechanical and chemical properties. This opens up a number of new perspectives including personalized medicine through the development of complex structures (the osteochondral compartment), different types of cartilage (hyaline, fibrous), and constructs according to a specific patient’s needs. However, the choice of the ideal combination of biomaterials and cells for cartilage bioprinting is still a challenge. Stem cells may improve material mimicry ability thanks to their unique properties: the immune-privileged status and the paracrine activity. Here, we review the recent advances in cartilage three-dimensional, scaffold-based bioprinting using stem cells and identify future developments for clinical translation. Database search terms used to write this review were: “articular cartilage”, “menisci”, “3D bioprinting”, “bioinks”, “stem cells”, and “cartilage tissue engineering”.

## 1. Introduction

Cartilage damages fail to heal spontaneously, leading to a progressive impairment of joint structure and function [1]. Articular cartilage is a hyaline tissue covering bone ends to enable free movements, as well as load bearing and distribution [2]. Its capacity for self-repair is low, resulting in the formation of a fibrous scar tissue with inferior mechanical and biochemical characteristics compared to native tissue. Joint tissues consequently tend to deteriorate with the following development of chronic disabling diseases, such as osteoarthritis (OA) [3]. Meniscus is a fibrocartilage structure providing shock absorption and cushioning in the knee [4]. Its peripheral vascularized region displays a healing potential, whereas the internal avascular region has low regeneration ability [5].

If left untreated, cartilage lesions worsen the life quality and work capacity of patients, resulting in enormous costs for health and social care systems [2]. Therefore, different options have been proposed over the years to treat cartilage damages, though they still represent a challenge for orthopedics. Palliative care, using pain-killers (analgesics) or anti-inflammatory drugs, may only alleviate articular cartilage symptoms without definitely solving the causes involved in the onset and progression of the disease [1,2]. Reparative strategies are able to fill the defect, but the neo-formed tissue has mostly fibrous features, hence favoring the development of OA and making joint prosthesis necessary [1,2]. Regenerative approaches including osteochondral grafting (autologous or allogeneic), and autologous chondrocyte implantation (ACI) are aimed at regenerating cartilage tissue with structural and functional features equivalent to native cartilage [1,2]. Despite encouraging results, these procedures still show shortcomings: donor site morbidity and graft failure due to peripheral chondrocyte death in the case of osteochondral grafting; implant availability and biomechanical integrity, short cell viability, and risk of disease transmission for allografts; cell leaking in the articular space and loss of chondrogenic phenotype in case of the ACI first-generation technique utilizing expanded chondrocytes; and the need for two interventions, delaying times and increasing costs for scaffold-based ACI [2]. As for meniscus, tissue tears can be treated with either non-operative treatments [6,7] or by surgical procedures such as partial or total meniscectomy. However, both interventions have been demonstrated to expose femoral condyles and tibial plateau to excessive wear, thus highly predisposing to OA [8]. Attempts to replace meniscus with cadaveric grafts (allografts) also display drawbacks: limited graft availability, pathogen transmission, immune rejection, and anatomical mismatch [8].

In the last decades, tissue engineering, intended as the combination of scaffolds, cells, and/or soluble/mechanical factors, has emerged as a promising strategy for cartilage regeneration [2]. The use of chondrocyte-loaded scaffolds was found to be effective in treating chondral and osteochondral defects and early OA lesions, reporting good clinical outcomes in terms of tissue healing, even if there are still issues due to limited cell availability and high costs for the expansion process [9,10]. Based on these limitations, researches have also considered alternative cells, such as mesenchymal stem cells (MSCs), characterized by a high proliferation rate and ability to self-renew and to differentiate towards several phenotypes, including the chondrogenic one [2,11,12]. Importantly, MSCs do not express molecules of class II major histocompatibility complex, essential for immune rejection, suggesting the possibility of an allogeneic use [2,11,12]. Recently, technological advances allowed the use of the entire bone marrow niche composed of MSCs and accessorized cells by avoiding laboratory manipulation (i.e., Bone Marrow Concentrate) [2,11,12]. Many studies have been conducted by using adult stem cells derived from different sources such as bone marrow, adipose tissue, muscles, synovial membrane, trabecular bone, dermis, blood, periosteum, and perichondrium. Perinatal/extra embryonic-associated tissues (i.e., amniotic fluid/Wharton’s jelly, umbilical cord, and placenta), embryonic, induced pluripotent, and genetically modified stem cells have also been investigated [13,14,15].

Although some reviews on cartilage bioprinting have already been published, we focused on the interactions between scaffolds and stem cells in forming bioinks suitable for translational research. Our aim is to evaluate the potential of three-dimensional (3D) bioprinting in improving cartilage tissue regeneration by exploring different combinations of biomaterials and stem cells. Finally, we considered possible future research development for cartilage regeneration in order to foster healing processes for a clinical setting.

## 2. Scaffold-Based Three-Dimensional Bioprinting for Cartilage Regeneration

3D bioprinting is a novel and innovative method for the 3D fabrication of living tissue/organ-like structures throughout the deposition of small units of a substance termed as “bioink” [2,16]. Two main bioink types have been recently developed: scaffold-based and scaffold-free. The former type is composed of cells and biomaterials which are released together in order to produce a construct. The material acts as a structural support or scaffold that creates an environment suitable for cell growth and differentiation [2,16]. The latter type is characterized by aggregates such as cell pellets, tissue strands, or spheroids which secrete extracellular matrix (ECM)-like structures holding together the cell component [17]. The scaffold-based approach is the first and most common, but both methods display advantages and limitations and it has been suggested that they can complement each other to help cover the broad spectrum of tissue engineering/regenerative medicine applications [18]. Here, we present new developments concerning the scaffold-based approach applied in cartilage tissue engineering, with stem cells as the living part.

### 2.1. The Technique

**Figure 1:**
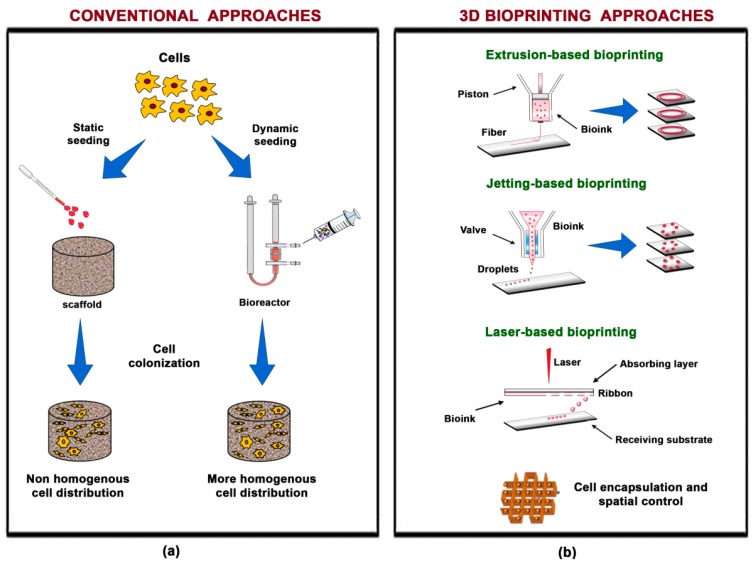
Comparison between tissue engineering conventional and three-dimensional (3D) bioprinting approaches. (**a**) In the conventional method, cells can be seeded in vitro in a static and/or dynamic mode onto a previously fabricated scaffold. The static seeding allows a non-homogenous cell distribution into the scaffold. The dynamic seeding can be carried out with different types of bioreactors to favor a more homogenous cell distribution. Here, there is an example of a perfusion bioreactor performing 3D cell seeding. Both types of “conventional” tissue-like constructs need to be subsequently shaped by the surgeon to match the defect site; (**b**) Representation of the main three bioprinting techniques: extrusion-based bioprinting; jetting-based bioprinting, and laser-based bioprinting. Such options allow cell encapsulation, spatial control, and the match between implant and defect size in the clinical setting.

Scaffold-based 3D bioprinting is a type of “additive manufacturing” whereby objects are built by the addition of subsequent, overlapping layers. As a consequence, constructs are composed from bottom to top [16]. Conversely, conventional “subtractive methods” remove materials from an initial block and generally depend on a top-to-bottom approach. The cell preparation is seeded onto the finished scaffold only at a later time (Figure 1a). Despite many efforts and improvements, such as dynamic seeding using bioreactors [19], conventional approaches still imply problems, due to the random cell distribution within the scaffold and, in particular, to the low density in the inner part where cells tend to die also because of the scarce concentration of nutrients in that area [19]. Bioprinting, owing to the intrinsic nature of the technique, displays the advantage of a fine control of cell spatial distribution, in terms of homogeneity, and of constructs composition and architecture by supporting good cell viability (Figure 1b). In particular, the improved possibility to modulate mechanical and chemical properties permits better mimicking of natural cartilage tissue characteristics and the creation of complex structures, such as the osteochondral compartment [2]. Further benefits from this technique include the reduced production times, an increased versatility, and the possibility to work under room temperature and “solvent-free” conditions. The bioprinting process, in fact, relies on the deposition of either cell-laden droplets or cell embedding in a hydrogel. Therefore, in most cases the technique does not require the use of toxic or harmful solvents for the cells, taking advantage of the features of water-based gels such as bioinks [16]. Importantly, 3D bioprinting belongs to a group of techniques, known as “rapid prototyping”, which allow the creation of objects by combining computer assisted design (CAD) with computer assisted manufacturing (CAM). Recently, the fabrication of custom-made products based on patient’s medical images acquired with non-invasive techniques such as Magnetic Resonance Imaging (MRI) and Computerized Tomography has become feasible [2,16]. Such options, improving the match between implant and defect size, can reduce the time required for surgery and for patient recovery, and positively affect treatment success. In general, the possibility to tailor therapy, also known as personalized medicine, is promising for improving health care and, at the same time, decreasing costs [20].

To date, the most used scaffold-based 3D bioprinting technologies are based on jetting, extrusion, and laser technology (Figure 1b).

Jetting-based 3D bioprinting can be performed as continuous inkjet or single droplets deposition with a specific distribution (drop-on-demand). Only the latter approach is considered suitable, since the former requires a conductive ink [14]. The drop-on-demand technique is based on three different mechanisms to generate droplets: piezoelectric, thermal, and electrostatic. Advantages of this technique are: low costs due to similarity with commercial printers, high printing speed as a consequence of the printer heads’ ability to support parallel work mode, and high cell viability (80–90%). Disadvantages are represented by narrow material selectivity and frequent clogging of the printer head [16]. To overcome existing issues and to obtain an improved performance, hybrid cell-printing techniques have been developed. Graham’s group discovered a bioprinting approach complementing the existing methodology for aqueous droplet printing in oil. In particular, they were able to print ovine MSC constructs submerged in oil that were subsequently encapsulated in a thin layer of gel for their following transfer to an aqueous medium. Incorporated cells displayed high viability (>95% average), proliferation ability, and the capacity to produce a cartilage-like ECM under transforming growth factor-β3 (TGF-β3) stimulation [21].

Extrusion-based 3D bioprinting has been already utilized to fabricate heterogeneous scaffolds for osteochondral regeneration. Most existing commercial bioprinters are based on this technology, which is able to dispense continuous filaments of bioink through a micro-nozzle using piston or pneumatic pressure. The advantages of this technique include the release of highly viscous bioinks with the micro-nozzle, the use of synthetic polymers, a scalable production, and a high cell viability. The main disadvantages are a relatively low resolution and deformation, and the apoptosis of encapsulated cells due to the great pressure generated along the nozzle by mechanical extrusion [14,16].

Laser-based 3D bioprinting is an expensive and complex technique that uses pulsed laser energy to transfer materials to a receiving substrate. Laser pulses are targeted on a gold layer of a ribbon, creating a high-pressure bubble, which drives the bioink towards a substrate. This is a nozzle-free procedure, and therefore it does not have clogging problems with cells or materials. Although this technique is able to produce higher resolution patterns, it results in lower cell viability in comparison to inkjet mechanisms [14].

### 2.2. Bioinks: Characteristics

Properties of bioinks are crucial for the development of functional living tissues by 3D bioprinting. Bioinks based on the combination of scaffold and cells should satisfy both biomaterial and biological features. Moreover, to mimic cartilage, specific features are required.

#### 2.2.1. Biomaterial Features

Printability, bioresorbability, and biodegradability are critical biomaterial requirements to guarantee a good performance.

Printability is the capacity to form a 3D structure as designed by CAD data. It is generally characterized by surface tension, viscosity, shear thinning, and cross-linking [22,23]. Surface tension regulation, by increasing bioink hydrophobicity or establishing an electrostatic interaction with the receiving surface, may increase the adhesion of the rising construct to the printing surface, avoiding any deformation or undesired movement [24]. Viscosity must be tuned to reduce the clogging of the printer head by allowing a liquid-phase, vertical, uninterrupted print flow and thus supporting the weight of the overlapped layers of the resulting structure [14]. Gao et al. developed a low viscosity bioink composed of acrylated poly(ethylene glycol) (PEG) hydrogel and human bone marrow-derived MSCs. Such a composition was shown to be able to minimize nozzle clogging, without affecting the quality of bioprinted bone and cartilage tissues in terms of ECM deposition and mechanical properties [25,26]. Shear thinning can be negatively influenced by a small nozzle diameter, as well as high printing pressure and viscosity. In the case of hydrogel bioink extrusion through a nozzle, viscosity decreases as shear rate increases [27,28]. Blaeser et al. investigated how shear stresses influence cell behavior in an alginate-based bioink printed by extrusion. They demonstrated that cell viability and proliferation were affected by the printing process, but not their phenotype and stemness [29]. Cross-linking is a strategy to improve bioink performance in terms of stability (each deposited layer should adhere to the previous one to create a solid, cohesive item), mechanical strength, modulus, and degradation properties. Therefore, bioinks should be cross-linkable and maintain this property after treatment [14,24], without affecting stem cell properties [30]. Cross-linking can be induced chemically, thermally, or using UV or visible light [24]. A classic example of chemical cross-linking is alginate with calcium chloride, a blend that allows stem cells to remain viable and metabolically active [31]. Among the various cross-linking reagents, glutaraldehyde, 1-ethyl-3-(3-dimethylaminopropyl) carbodiimide hydrochloride, and genipin are partially used because of their potential toxicity, especially for glutaraldehyde [14]. Genipin, an extract from the gardenia fruit, is considered the least cytotoxic, although it has relative high costs. Enzymatic cross-linking reactions (mushroom tyrosinase and microbial transglutaminase) take place typically in conditions that are favorable to cells i.e., neutral pH, aqueous solution, and physiological temperatures [14]. Das et al. utilized tyrosinase to cross-link a silk fibroin-gelatin hydrogel and observed that embedded MSCs were still able to differentiate towards chondrocytes [32]. Thermal cross-linking has been successfully utilized to switch hydrogels based on poly(*N*-isopropylacrylamide) from a water-swollen state at low temperature to a collapsed state at high temperature [33]. Hydrogel cross-linking by a light source is an interesting approach that exploits material absorbance properties. Initiators such as 2-hydroxy-1-[4-(hydroxyethoxy)phenyl]-2-methyl 1-propanone and lithium phenyl-2,4,6-trimethylbenzoylphosphinate are utilized to improve photosensitivity. UV light has been utilized to stabilize nanostructured hydrogels derived from the combination of acrylate and pluronic. The cross-linking treatment increased encapsulated cell viability at day 14, from 62% to 86%, at the same time maintaining the printability of pluronic. However, there are still problems to solve, such as the formation of free radicals that can negatively influence cell viability [34,35].

Bioresorbability and biodegradability are other critical properties that allow scaffold degradation within the implantation site at the same rate of cell colonization [36].

Other important characteristics of bioinks include: high resolution, low cost, industrial scalability, short post-printing time for maturation and, in the case of hydrogels, reversibility of gelation (relevant for pre-culture before delivery), fast gelling, and the absence of volume change during gelling [24].

#### 2.2.2. Biological Features

Biocompatibility with living systems is a basic feature common not only for a bioink, but also for all biomaterials utilized in tissue engineering. Other important biological “non-properties” of materials are to be non-toxic, non-immunogenic, non-thrombogenic, and non-carcinogenic [37].

Cytocompatibility allows the preservation of the biological features of encapsulated cells in terms of viability, proliferation, function, and ECM production. In general, cell integrity is implemented by using water soluble materials, such as hydrogels, which are permeable to oxygen gas, nutrients, and catabolic products [36,37].

Bio-inertness should be regulated since it may not only negatively influence cell capacity to proliferate and move within the bioink, but also elicit death through anoikis (apoptosis induced by cell detachment from the ECM) [38]. To overcome this issue, the development of a mixture of a hyaluronic acid (HA) hydrogel with dextran with enhanced protein-adhesion characteristics was proposed. This resulted in a higher viability of bioprinted adipose-derived stem cells (ASCs) [14].

#### 2.2.3. Cartilage Tissue Engineering Characteristics

Regeneration ability is crucial for cartilage tissue engineering applications. In particular, the neo-synthesis of ECM cartilage can be induced by stem cells properly stimulated by specific soluble factors absorbed within the bioink, such as TGF-β1 and bone morphogenetic protein-6 (BMP-6), or added to the culture medium, such as TGF-β3, sodium pyruvate, dexamethasone, insulin, transferrin, selenous acid, and ascorbate-2 phosphate (chondrogenic medium) [14]. It has been demonstrated that human ASCs embedded in gelatin-based hydrogels and cultured in the presence of a chondrogenic medium produced an ECM with typical cartilaginous features [39]. Soluble factors can also be utilized to trigger specific zonal phenotypes which are typical, for instance, of the osteochondral compartment or the meniscus. Romanazzo et al. were able to reproduce the two meniscal regions by bioprinting infrapatellar fat pad-derived stem cells with alginate hydrogels. They utilized two different growth factors in the two different zones: TGF-β3 to induce the synthesis of collagen type II and sulfated glycosaminoglycans, typical of the inner (avascular/aneural) region, and connective tissue growth factor (CTGF) to induce a more fibrogenic phenotype characteristic of the outer (vascular/neural) region [40].

Mechanical features of bioprinted constructs are inferior to those of the native tissue. For hydrogels, this is mainly due to the random alignment of fibres and the high water content [37]. Several strategies can be applied to strengthen mechanical performance: supplementation with mineral particles (e.g., hydroxyapatite), cross-linking, and hybrid/composite creation (i.e., reinforcement with poly(ε-caprolactone) (PCL) [24,37]. Stichler et al. attempted to mimic articular cartilage features by printing a thiol-functionalized HA (HA-SH) hybrid hydrogel embedded with human and equine MSCs. For the cross-linking process they utilized allyl-functionalized poly(glycidol)s (P(AGE-co-G)). To achieve more mechanically stable and robust constructs, double printing with thermoplastic PCL was employed. Double-printed gels are characterized by two types of components with opposite physical natures; the minor is soft and the major is tough. This makes them unique, since they acquire greater mechanical strength and toughness, still maintaining a high water content. For instance, in a swine model they were able to reproduce meniscus dynamic stiffness [41,42].

### 2.3. Bioinks: Current Options

Generally, available scaffold options for bioinks are hydrogels, decellularized ECM (dECM), or microcarriers.

#### 2.3.1. Hydrogels

Hydrogels are the most commonly used bioinks, due to their swelling and lubricating characteristics that best match with cartilage properties, and their potential to induce cells towards a chondrogenic phenotype [43,44]. They are semi-liquid hydrophilic colloids composed of a network of natural or synthetic cross-linked polymer chains. The gel consistency allows easy casting into the desired shapes and a uniform mixing with cells and growth factors. High porosity helps cell diffusion, but it has poor mechanical properties by failing to recapitulate the biological feature of cartilage with no proper functionality [14,24,45]. To overcome such an issue, cross-linking agents such as glutaraldehyde have been used by various scientists reporting the decreased immunogenicity of implants and an increase in cytotoxicity [7]. As previously mentioned, double-network hydrogels can be a solution. Other disadvantages of hydrogels are the limited critical timing of gelation, specific matching of material and liquid medium densities, and low resolution [7,44,45].

Depending on their origin, hydrogels can be natural or synthetic. Natural hydrogels used for cartilage tissue engineering include agarose and alginate, collagens and gelatins, HA, chitosan, gellan gum, fibroin silk proteins, cellulose, and fibrin [16], which display a good biocompatibility and biodegradability [45,46].

Agarose, a marine polysaccharide obtained from seaweed, is one of the first hydrogels developed for cartilage tissue engineering applications. This is due to specific features such as easy gelation, excellent biocompatibility, the ability to support the chondrogenesis of MSCs, and a stiffness and viscoelasticity similar to native cartilage [47,48].

Alginate is a negatively charged polysaccharide extracted from brown algae, which does not elicit inflammatory response when implanted in vivo. The most common calcium chloride cross-linked alginate-based hydrogels present some advantages such as biocompatibility for MSCs and low toxicity [49,50].

Collagens, the main protein components in natural cartilage, have a documented use as components of scaffolds for cartilage tissue engineering [2]. Collagen-based hydrogels, displaying great biocompatibility and biodegradation without causing inflammation, have been demonstrated to favor MSCs adhesion and chondrogenesis [51,52,53]. A main issue, represented by the poor mechanical properties, can be overcome by cross-linking [54,55].

Gelatins, derived from collagen partial hydrolysis, are characterized by cell adhesive properties, low antigenicity and immunogenicity, but poor stability. Generally, chemical cross-linking or hybrid materials can increase stability, but they could determine toxic effects [56,57]. Gelatine methacrylamide (GelMA) has gained increasing attention for cartilage tissue engineering applications due to its ability to mimic ECM and its tunable physical characteristics [58].

HA is an anionic, non-sulfated glycosaminoglycan which is present in cartilage ECM, synovial fluid, and stem cell niche, supporting cell attachment through surface receptors like CD44 [2,59]. It has been widely utilized in tissue engineering applications to promote cartilage formation [60,61]. Recent in vitro and in vivo preclinical studies have shown the possibility to create HA-based bioinks whereby embedded stem cells are able to maintain their multipotency [62] and, more importantly, can differentiate towards the chondrogenic phenotype [63,64].

Chitosan is one of the most abundant aminopolysaccharide polymers derived from chitin (shell of shellfish, crustacean shells, insect cuticles, mushroom envelopes, and the wastes of the seafood industry [65]) through deacetylation. It has already been demonstrated to promote cartilage formation, alone or in combination with other biomaterials such as HA, which enhances its properties [2,16]. Chitosan is biocompatible and biodegradable. Enzymatic cross-linking can improve MSC proliferation and chondrogenic differentiation, both in vitro and in vivo, as shown by preclinical studies [66,67,68]. Drawbacks are poor solubility in water [69], low cellular adhesiveness, and potential allergenic reactions due to its origin [70].

Gellan gum is an anionic microbial polysaccharide already used in the food, pharmaceutical, and medical industries [71]. Through thermally reversible gelation processes, hydrogels can be formed [51]. Gellan-based materials have been recently proposed in regenerative medicine, particularly in cartilage tissue engineering [72]. Issues for bioprinting are represented by a gelation temperature (>42 °C) higher than the physiological temperature, which may compromise cell viability and cause the loss of stability and mechanical properties [73,74].

Fibroin silk, produced from silkworm (Bombyx mori) cocoons [75], appears promising for cartilage bioprinting as it is easily available, biocompatible, biodegradable, mechanically robust, and able to induce the synthesis of cartilage ECM by MSCs [76,77]. Issues consist in a low concentration range (<20 wt %) which is not optimal for printing, and in the possible degradation of the fibroin protein chains during silk fibroin processing [78].

Cellulose, one of many polymers found in nature, may enter the composition of carboxymethyl cellulose, and in turn, hydrogel [36], by specific processes. Recently, it has been tested for the bioprinting of cartilage [79,80].

Fibrin is a blood protein, well known for its role in clot formation, justifying its use in clinical practice as a hemostatic or a sealant agent. Hydrogels can be prepared from fibrinogen by the enzymatic treatment of thrombin; the advantages are excellent biocompatibility and biodegradability, but weak mechanical properties [81]. However, their combination with particulate ECM may enhance the chondrogenic potential [36].

Synthetic biocompatible polymers already utilized to develop hydrogels for cartilage tissue engineering include poly(vinyl alcohol) (PVA), PEG, and pluronic [36,45]. Their properties can be custom-designed in a more controllable manner than natural hydrogels; however, their hydrophobicity may compromise their biocompatibility [2,36,45]. The possibility to blend together natural and synthetic hydrogels may reduce problems derived from each single component while also exceeding the results obtained when the materials are used separately.

**Figure 2:**
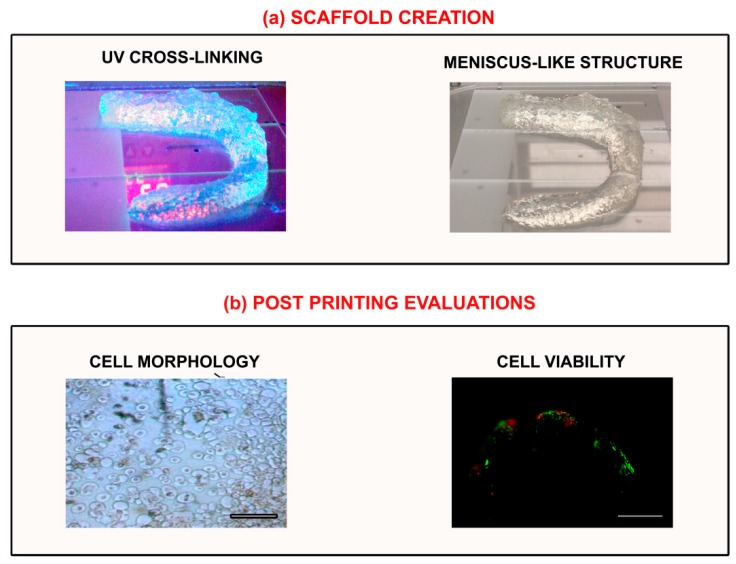
(**a**) Custom-made printed meniscal model. The construct is realized in a poly(ethylene glycol) methacrylate (PEGMA)-based hydrogel (Bioink, RegenHU, CH) that requires UV light cross-linking (365 nm wavelength) to improve structural and mechanical properties; (**b**) Post-printing evaluations: mesenchymal stem cell (MSC) distribution by microscopy and cell viability by the Live and Dead test (green: live cells (FITC channel); red: dead cells (TRITC channel); scale bar: 100 µm).

PVA- and PEG-based hydrogels have been developed in a variety of cartilage tissue engineering studies; however, the first is not biodegradable and can only be used for permanent cartilage implants [82,83]. PEG methacrylate (PEGMA)- and diacrylate (PEGDA)-based hydrogels can be instead improved by a cross-linking process to induce MSC chondrogenic differentiation [84]. In our laboratory, we have developed a PEGMA hydrogel that mimics the meniscal structure. In order to increase the structural support ability, the hydrogel was UV cross-linked and the MSCs were encapsulated. Homogeneous cell distribution and high cell viability were noticed following post-printing processes, indicating biocompatibility features. However, further optimization is needed to ameliorate the mechanical properties typical of meniscus (Figure 2).

Pluronics, also known as poloxamers, have surfactant properties which allow them to interact with hydrophobic surfaces and biological membranes [36,45]. Thermo-sensitive chitosan-pluronic hydrogels have been demonstrated to be able to support chondrocyte growth [85]. Their combination with acrylate [35] allows the development of a nanostructured hydrogel showing printability characteristics and, at the same time, increased cell viability.

Recently, the number of studies focused on the development of hydrogel-based scaffolds has increased and some research groups have tried to confront their healing potential in terms of tissue regeneration quality. Costantini et al. compared different scaffolds: GelMA, GelMA + chondroitin sulfate amino ethyl methacrylate (CS-AEMA) and GelMA + CS-AEMA + HA methacrylate (HAMA). All hydrogels were combined with alginate as a templating agent to form stable fibres, printed with human bone marrow-derived MSCs, and cultured in chondrogenic medium for three weeks. The presence of collagen types I, II, X and aggrecan was evaluated qualitatively, by immunocytochemistry, as well as quantitatively by quantitative real time Reverse Transcription Polymerase Chain Reaction (RT-qPCR) analyses. Immunocytochemical results did not show differences between the three scaffolds. Differently, real time RT-qPCR analyses showed that the CS-AEMA-based scaffold was the best candidate in improving the regeneration of hyaline cartilage expressing 6-fold more collagen type II than collagen type X. Differently, the HAMA-based scaffold favored the differentiation of MSCs to a hypertrophic phenotype with a high expression of collagen type X, thus predisposing to OA onset [86]. This last result was in accordance with previous data highlighting that increasing HA concentration worsens tissue quality, with a clear shift towards the hypertrophic differentiation of the loaded MSCs [87].

**Figure 3:**
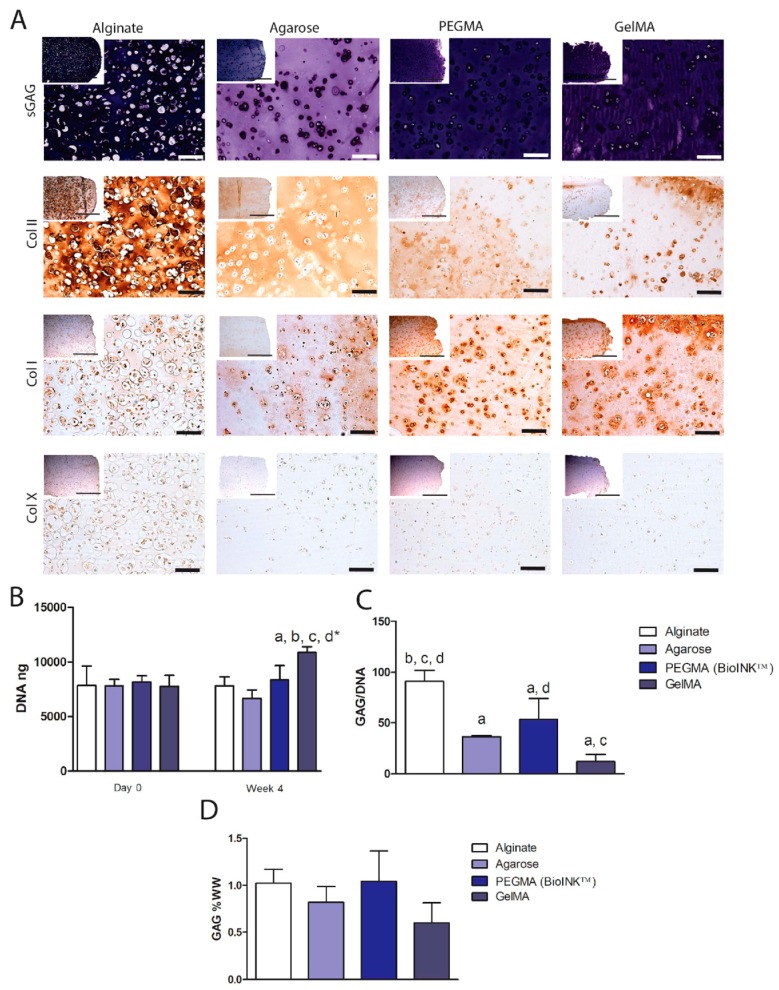
Regenerative ability of agarose-, alginate-, GelMA- and PEGMA-based hydrogels seeded with MSCs and cultured in a chondrogenic medium for four weeks. (**A**) Immunohistochemical and histological analyses showed that agarose and alginate both stained for collagen type II and glycosaminoglycans (GAG). In contrast, GelMA and PEGMA were more positive collagen type I; (**B**) DNA content (ng) quantification for each construct; (**C**) GAGs per DNA ratio; (**D**) Weight/weight GAG percent (%WW). Biochemical analyses showed that GelMA had the lowest GAG levels. Significance *p* < 0.05, (a) versus alginate at the same time point, (b) versus agarose, (c) versus PEGMA, (d) versus GelMA, (d*) versus GelMA at day 0 [88]. Reproduced with permission from IOP SCIENCE.

Daly et al. analyzed four commonly used hydrogels, two natural and two synthetic: agarose-, alginate-, GelMA-, and PEGMA-based scaffolds. After seeding with bone marrow-derived MSCs and culturing in a chondrogenic medium for four weeks, bioinks were compared for their differentiation potential towards hyaline cartilage or fibrocartilage phenotype by in vitro studies. Findings from this study showed that alginate and agarose hydrogels provide a good support to develop a hyaline-like tissue with a high content of collagen type II, while GelMA- and PEGMA-based bioinks sustained the formation of fibrocartilage, typically composed of collagen type I. These data provided important clinical insights, since they demonstrated that different scaffolds may be suitable for different orthopedic applications, and highlighted the importance of selecting the correct bioink according to the clinical needs. In particular, alginate and agarose hydrogels seemed to be suitable for articular cartilage repair strategies, while GelMA- and PEGMA-based bioinks appeared to be indicated to treat meniscal tears [88] (Figure 3).

#### 2.3.2. Decellularized ECM

dECM can be obtained from tissue trypsinization followed by washing. Such a procedure preserves the ECM components, while removing cells, debris, and immunogenic material. The drawbacks of this technique are the presence of chemical residuals and an important loss or damage of matrix components. The obtained dECM can be directly utilized for tissue engineering applications by cell colonization or further treated until lyophilization to powder to produce hydrogels [89].

#### 2.3.3. Microcarriers

A microcarrier is a polymer matrix with microspheres typically ranging in size from 60 to 400 μm, whose surface serves as a support for MSCs to form multi-cellular aggregates [90,91]. Surface area and composition can be modified in order to increase adhesion properties or trigger stem cell differentiation. In the work by Levato et al., MSC-laden polylactic acid (PLA) microcarriers encapsulated in a gelatin methacrylamide-gellan gum bioink were demonstrated to support cell viability and growth during bioprinting. Importantly, the MSCs were able to differentiate towards osteogenic and chondrogenic phenotypes, thus mimicking the osteochondral compartment [92].

## 3. Future Developments

Due to its characteristics, 3D bioprinting has had, from its introduction in the medical field, a great impact on researchers and clinicians, and it has also raised numerous expectations in the public opinion. Nevertheless, this technology is still in its early days and many issues must be addressed prior to translation into clinical routine, including economic and ethical obstacles. In the specific case of cartilage, such tissue would seem easier to print, because of its thickness, avascularity, and scarce cellular component, especially when compared to other tissues including bone, which requires the manufacture of a proper vascular network for its viability and tissue engraftment. However, it is well known that cartilage seemingly displays a simple structure. In fact, it is characterized by a zonal architecture defining specific mechanical properties which are very difficult to reproduce artificially [1,4].

**Figure 4:**
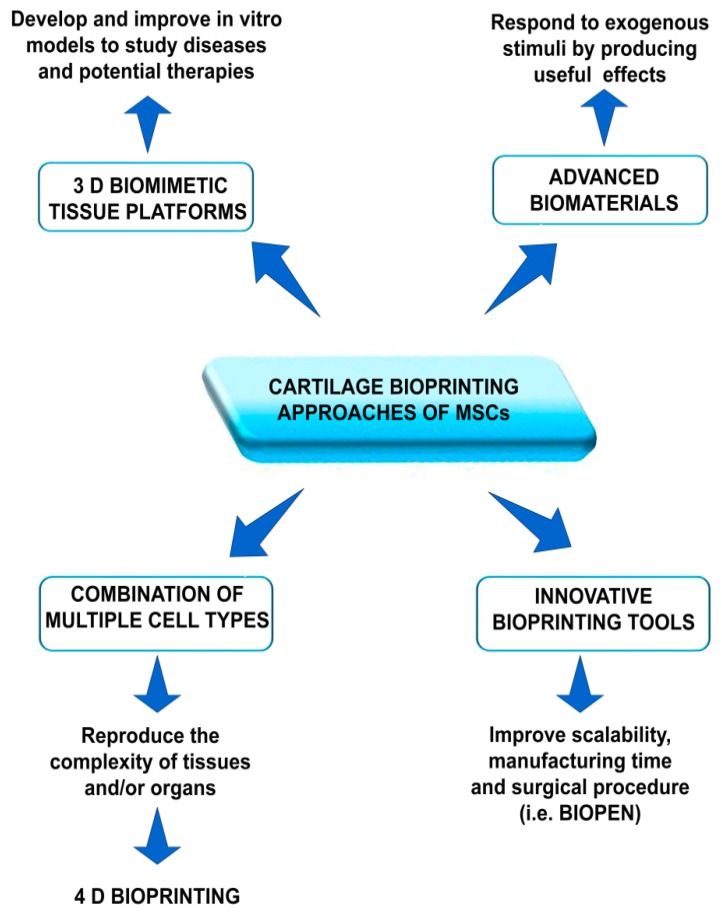
Schematic representation of four possible future perspectives for cartilage bioprinting approaches of MSCs. Upper left part: 3D biomimetic tissue platforms for the development of micro-organs/-tissues as new models for mimicking diseased anatomical sites and studying possible therapies (i.e., organ-on-chip); upper right part: advanced biomaterials that can modify their properties according to biological cues: 4D bioprinting; lower left part: combination of multiple cell types to mimic the tissue complexity; lower right part: innovative bioprinting tools to improve scalability, manufacturing time, and surgical approach (i.e., Biopen).

Further pre-clinical in vitro and in vivo studies are needed for a more rapid clinical translation of this technology, to satisfy patient’s needs [92]. To reach these goals, we believe that there will be progresses aimed at developing innovative biomimetic tissue platforms suitable for more in-depth in vitro testing as well as improving scaffold performance—for example, the production of advanced, smart materials by four-dimensional (4D) printing (Figure 4).

Stem cells seem to be good candidates for cartilage bioprinting due to their already described properties [11,12,93], but there are some limitations. A main issue is represented by the regenerative ability towards the desired cartilage phenotype (hyaline or fibrous), and thus a functional tissue. For instance, MSCs display the tendency to progress into a hypertrophic phenotype and thus giving rise to endochondral bone formation [11,12,93]. Therefore, the search for more effective approaches avoiding hypertrophy is demanding. To this end, Gao et al. investigated the role of nuclear receptor subfamily 2 group F member 2 (NR2F2) in PEGDA/MSC-based bioprinted cartilage-like constructs. They observed that NR2F2 lentivirus over-expressing MSCs showed significantly enhanced chondrogenesis, in terms of matrix composition and mechanical features, exhibiting an opposite behavior compared to that of NR2F2 siRNA knockdown MSCs [26]. In the light of these considerations, we acknowledge the possibility to use multiple cell types as a valid future way to improve the regeneration process, better mimicking cartilage tissue structural and functional complexity (Figure 4).

Finally, further improvements in the printing devices will be mandatory in the near future in order to be transportable in the operating room and easily usable by surgeons (Figure 4).

### 3.1. Biomimetic Tissue Platforms

The integration of 3D bioprinting into a biomimetic model or organ-on-a-chip engineering can facilitate (i.e., high accuracy, high-throughput assay) the creation of micro-organs or -tissues with specific features. These structures can represent suitable tools for in vitro testing or serve as a valuable intermediate for 3D tissue engineering [94].

3D biomimetic tissue models have recently emerged and started playing an important role in drug discovery and development, thereby reducing the need for animal testing. A significant challenge is represented by the ability to engineer multiphase complex structures such as tissue interfaces. Gurkan et al. developed a microscale model mimicking a gradient fibrocartilage tissue via the encapsulation and printing of human MSCs with BMP-2 and TGF-β1. Results suggested the simultaneous differentiation of MSCs towards osteogenic and chondrogenic lineages within the multiphasic construct [95].

Organ-on-a-chip technology promotes the formation of tissue- or organ-like structures on a microchip platform. Such systems can be developed by using dynamic fluid flow to produce nutrition, oxygenation with tissue-specific environmental cues, and molecular gradients. Environmental physical factors such as pH, temperature, oxygen concentration, and humidity are controlled. One advantage of this technology is the possibility to mechanically or biochemically change the chip setting, thus reproducing pathological conditions, such as cartilage degradation in OA. This can provide insight on both the intricate nature of diseases and their treatments [69,96].

### 3.2. Advanced Materials

Tissues are plastic, having dynamic changes in conformation and according to their functional status and environmental stimuli. It would be important to control the bioink geometry, porosity, and cell distribution, not only during the bioprinting process, but also post-printing, in order to better drive the regeneration process [97]. One valid opportunity is 4D printing. This is an extension of 2D and 3D printing in which the printed structure transforms or reshapes post deposition, in response to external stimuli (temperature, pH, light, electricity etc.) [98]. Stem cell ability to differentiate can dramatically alter the bioactivity, biodegradation, and mechanical properties of the bioprinted construct; hence, it may be considered as a form of 4D bioprinting [14]. Although not yet referred to cartilage, some examples have been found in the literature. In the paper by Rutz and colleagues, human MSCs were cultured onto a bioprinted PEG constructs with a grid pattern for two weeks. As a result, matrix deposition rendered the bioink more robust and prolonged the degradation time [46].

### 3.3. The Use of Multiple Cell Types

In general, tissues show heterogeneity in terms of cell types and distribution. The possibility to create tissues using multiple cell types represents a new opportunity in terms of biomimicry, also exceeding the results obtained when they are used separately. In fact, despite various attempts to generate cartilage with chondrocytes or MSCs used individually, recently the combination of both cell types has been demonstrated to enhance chondrogenesis. In the work by Apelgren et al., mono- and co-cultures of human chondrocytes and bone marrow-derived MSCs were printed in a nano-fibrillated cellulose (NFC)/alginate hydrogel. Constructs were then implanted subcutaneously in nude mice and explanted after 30 and 60 days for morphological and immunohistochemical examinations. As a result, enhanced proliferation and chondrogenesis were observed, compared to constructs carrying the two cells separately [99]. Möller et al. performed similar experiments, confirming previously obtained results. Both studies support the hypothesis that the mechanism associated with the proliferative effect of stem cells on chondrocytes is related to paracrine signaling [100].

Levato et al. compared the ability to produce cartilage in GelMA-based hydrogels of multipotent articular cartilage-resident chondroprogenitor cells (ACPCs), MSCs, and chondrocytes. ACPCs, mainly located in the superficial zone of articular cartilage, may be considered interesting for their important role in tissue development, similar to that of MSCs. Results highlighted that APCs showed the lowest gene expression level of the hypertrophy marker collagen type X, and the highest expression of PRG4, a key factor within the superficial zone involved in joint lubrication. Such data induced the authors to create bioprinted cartilage constructs with MSCs in the middle/deep layer and ACPCs on the surface. Histological analysis confirmed a zonal difference in the distribution of proteoglycans, from the ACPC-laden to the MSC-laden zone, similar to that of articular cartilage [101].

Nguyen et al. printed human Induced Pluripotent Stem Cells (iPSCs) and chondrocytes within two different NFC compositions: alginate or HA. Low cell proliferation and phenotypic changes were observed following the use of NFC/HA. In the case of NFC/alginate, pluripotency was initially maintained, but after five weeks a differentiation pathway was undertaken, as demonstrated by the formation of hyaline-like cartilaginous areas positive for collagen type II and lacking Octamer-binding transcription factor 4 (Oct4) expression. Tumorigenic protein Oct4 can be considered as the primary factor regulating self-renewal and differentiation. Its decrease is important in a clinical setting because it indicates the reduction of pluripotency, which increases the risk of potential tumor formation [79].

### 3.4. Bioprinting Tools Suitable in the Operating Room

One of the mostly recognized advantages of 3D printing in medicine is the production of custom-made products. However, at this stage, the only solution is to create the final product in the laboratory and subsequently transfer it to the patient. Moreover, for clinical use, the bioprinted implants have to meet safety and quality requirements [102]. However, there are not many automated closed systems suitable for this purpose. Most commercial printers nowadays work on an open-loop format and have no feedback on the quality of the print, thus requiring monitoring from skilled operators [103].

In situ techniques, where the bioink is directly bioprinted into lesion sites, have been demonstrated to be reliable in animal models for the treatment of bone defects [104]. However, when translating to humans, the printing device is still too cumbersome to be used in a surgical setting. A printing system that could be used directly by the surgeon would avoid laboratory-based passages, and circumvent the need for two surgical interventions, with better compliance for the patient and faster recovery time [105].

O’Connell et al. published a recent work which goes in the direction of translating the 3D bioprinting procedure into a surgical process for chondral defect repair. In particular, they developed a handheld fabrication tool, termed a ‘biopen’, which enables the deposition, through custom titanium nozzles, of a bioink composed of human ASCs and GelMa/HAMa in a manual, direct-write procedure. In vitro experiments demonstrated not only that cells maintained a high viability (>97%), but also that the act of bioprinting did not elicit any visible effect. Finally, the authors stated that fabrication parameters still need to be optimized, especially concerning the shear stress experienced by cells during the extrusion and the cytotoxicity rate due to the cross-linking procedure [106]. To overcome these and other challenges, the same group improved the tool by using what they termed the core/shell principle of deposition in further studies. In particular, the pen was able to depose a bioink that was composed of two components: an inner core containing ASCs encapsulated in a GelMa/HAMa hydrogel (10%/2%) and an outer shell carrying the same hydrogel, but photo-cross-linked. Hardening of the shell provided structural properties allowing 3D printing, while cells were preserved in a relatively soft, comfortable environment inside the core. According to the authors, this approach could lead the way to the use of materials that are precluded on the basis of their inherent toxicity issues [107].

## 4. Conclusions

3D, scaffold-based bioprinting has the potential to reproduce both articular cartilage and meniscal structures. Advantages of the above conventional fabrication methods consist in their precise spatial control and individual design. However, several hurdles must still be overcome before clinical applicability is achieved. In particular, technical challenges concern bioink formulation and technique amelioration and standardization. In the case of cartilage, a significant challenge is represented by the difficulty to mimic mechanical properties.

Stem cells, already demonstrated to be valuable mediators for tissue regeneration, are also promising candidates for this technology. When associated with different biomaterials and soluble factors within bioink, they seem to maintain viability and enhance the chondrogenic process.

3D bioprinting has a great impact on public opinion and raises numerous expectations. It is desirable that this will positively push research activity and therefore accelerate the translation into clinical practice in the near future.
